# Characterizing the Effects of Washing by Different Detergents on the Wavelength-Scale Microstructures of Silk Samples Using Mueller Matrix Polarimetry

**DOI:** 10.3390/ijms17081301

**Published:** 2016-08-10

**Authors:** Yang Dong, Honghui He, Chao He, Jialing Zhou, Nan Zeng, Hui Ma

**Affiliations:** 1 Shenzhen Key Laboratory for Minimal Invasive Medical Technologies, Institute of Optical Imaging and Sensing, Graduate School at Shenzhen, Tsinghua University, Shenzhen 518055, China; dy15@mails.tsinghua.edu.cn (Y.D.); he.honghui@sz.tsinghua.edu.cn (H.H.); he-c13@mails.tsinghua.edu.cn (C.H.); zhoujl14@mails.tsinghua.edu.cn (J.Z.); zengnan00@mails.tsinghua.edu.cn (N.Z.); 2 Department of Biomedical Engineering, Tsinghua University, Beijing 100084, China; 3 Department of Physics, Tsinghua University, Beijing 100084, China

**Keywords:** silk, microstructure, polarization, Mueller matrix

## Abstract

Silk fibers suffer from microstructural changes due to various external environmental conditions including daily washings. In this paper, we take the backscattering Mueller matrix images of silk samples for non-destructive and real-time quantitative characterization of the wavelength-scale microstructure and examination of the effects of washing by different detergents. The 2D images of the 16 Mueller matrix elements are reduced to the frequency distribution histograms (FDHs) whose central moments reveal the dominant structural features of the silk fibers. A group of new parameters are also proposed to characterize the wavelength-scale microstructural changes of the silk samples during the washing processes. Monte Carlo (MC) simulations are carried out to better understand how the Mueller matrix parameters are related to the wavelength-scale microstructure of silk fibers. The good agreement between experiments and simulations indicates that the Mueller matrix polarimetry and FDH based parameters can be used to quantitatively detect the wavelength-scale microstructural features of silk fibers. Mueller matrix polarimetry may be used as a powerful tool for non-destructive and in situ characterization of the wavelength-scale microstructures of silk based materials.

## 1. Introduction

Silk based materials have been applied to many areas such as medicine [1], biotechnology [2,3], biomaterials [4,5], fine chemical industry [6] and so on. Silk consisting of fibroin and sericin may suffer from microstructural changes due to hostile external environmental conditions such as PH, temperature, or exposure to ultraviolet light [7,8,9,10]. In our daily life, silk fabrics are often washed using different detergents, which may have a different influence on the microstructure of silk. Recently, many techniques have been used to study the physics and chemistry properties of silk fibers, such as X-ray diffraction, scanning electron microscopy, nuclear magnetic resonance, infrared spectroscopy, Raman spectroscopy and so on. It will be useful if we have non-destructive and real-time techniques to quantitatively monitor the structural variations of silk based materials under different conditions.

Mueller matrix polarimetry has been recognized as a potentially powerful technique for probing the wavelength-scale microstructural and optical properties of complex scattering samples [11,12]. Mueller matrix imaging techniques have many distinctive advantages for non-invasive and in-situ applications on biological specimens or other delicate samples [13,14,15,16]. Most of the existing non-polarization optical instrumentations can be upgraded for Mueller matrix measurements by adding proper polarization state generator (PSG) and polarization state analyzer (PSA) to the existing optical path [17]. Comparing with non-polarization measurements, a Mueller matrix contains much richer wavelength-scale microstructural information, which can be used to characterize the structural features of the scattering media, such as polymer materials, and biomedical samples [14,15,16,17]. Mueller matrix polarimetry may also serve as a powerful tool to distinguish the characteristic features of different textile samples, such as acetate, cotton, silk and ramie [18]. In recent studies, Mueller matrix imaging parameters have been used to provide comprehensive characterization of the polarization features to assist various pathological detections, such as skin cancer [19], cervical cancer [20], colon cancer [21], liver fibrosis [22], and so on [23,24].

In this paper, we use Mueller matrix polarimetry for quantitative characterization of wavelength-scale microstructures of silk samples. The silk samples are washed several times using different detergents, such as fabric softener, laundry powder, toilet soap and color stain net. The backscattering Mueller matrix images are taken after each washing process. The 2D images of the 16 Mueller matrix elements are then reduced to their frequency distribution histograms (FDHs) and central moments. According to the features of silk fibers, we also propose new parameters based on the central moments of FDHs to quantitatively characterize the wavelength-scale microstructural changes of the silk samples washed by different detergents. For a deeper understanding of the relationship between the Mueller matrix parameters and the microstructural variations of silk, we also perform the X-ray diffraction and scanning electron microscope measurements then carry out Monte Carlo (MC) simulations based on the sphere-cylinder scattering model (SCSM) [25]. The good agreement between experiments and simulations indicates that the Mueller matrix imaging and FDH based parameters can be used to obtain quantitatively the wavelength-scale microstructural features of silk samples. Mueller matrix polarimetry may be used as a powerful tool for non-destructive and real-time characterization of the wavelength-scale microstructures of silk based materials.

## 2. Results and Discussion

### 2.1. 2D Images of Mueller Matrix Elements

**Figure 1:**
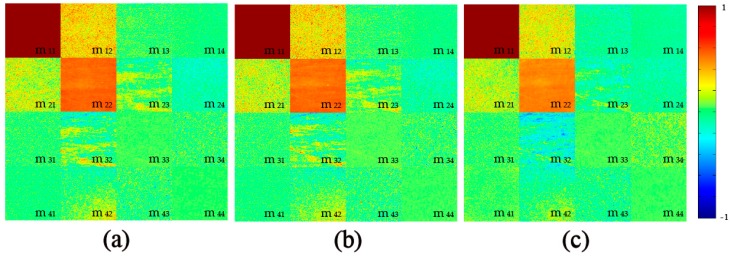
2D images of Mueller matrices of silk sample washed by fabric softener: (**a**) before washing; (**b**) after the third washing; (**c**) after the sixth washing. The color bar is from −1 to 1 for the m11, m22, m33, and m44, and from −0.1 to 0.1 for other elements.

**Figure 2:**
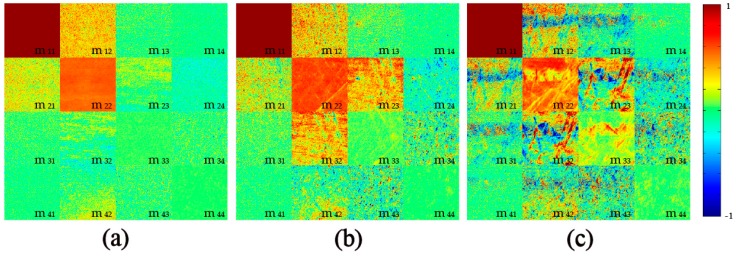
2D images of Mueller matrices of silk sample washed by color stain net: (**a**) before washing; (**b**) after the third washing; (**c**) after the sixth washing. The color bar is from −1 to 1 for the m11, m22, m33, and m44, and from −0.1 to 0.1 for other elements.

Figure 1 and Figure 2 show the experimental results of 2D Mueller matrix images for the silk samples washed by fabric softener and color stain net, respectively. All the matrix elements are normalized by the m11. As we have learned from previous studies [20,26], the following structural and optical information of the silk samples before and after washing can be obtained from the Mueller matrix elements: First, in Figure 1 and Figure 2, the differences between the diagonal m22 and m33 elements, and the magnitudes of the off-diagonal elements indicate that the silk sample is highly anisotropic. Experimental and simulated results have demonstrated that a larger difference between the m22 and m33 means a more prominent anisotropy [20,26]. It can be observed from Figure 1 that with more washing time using fabric softener, the Mueller matrix of the silk sample remains almost the same, while Figure 2 shows that the washing process using color stain net significantly changes the Mueller matrix: the decreasing difference between the m22 and m33 indicates that the well-ordered anisotropic silk fibers may be destroyed. Second, in Figure 1a and Figure 2a the m12, m21 elements are both positive, while the m13, m31 elements are approximately zero, which demonstrates that the orientation of the silk fibers is mostly along the 0 degree direction [12]. Figure 2b,c show that the orientation of the silk fibers washed by color stain net becomes disordered. Third, the diagonal elements m22, m33 and m44 represent the depolarization ability [26]. Figure 1 shows that when washed by fabric softener, the depolarization ability of the silk samples almost remains constant, while in Figure 2 the decrease of the diagonal elements indicates that the depolarization ability of the silk samples increases as the washing times using color stain net rise.

### 2.2. Frequency Distribution Histogram (FDH) of Mueller Matrix Elements

**Figure 3:**
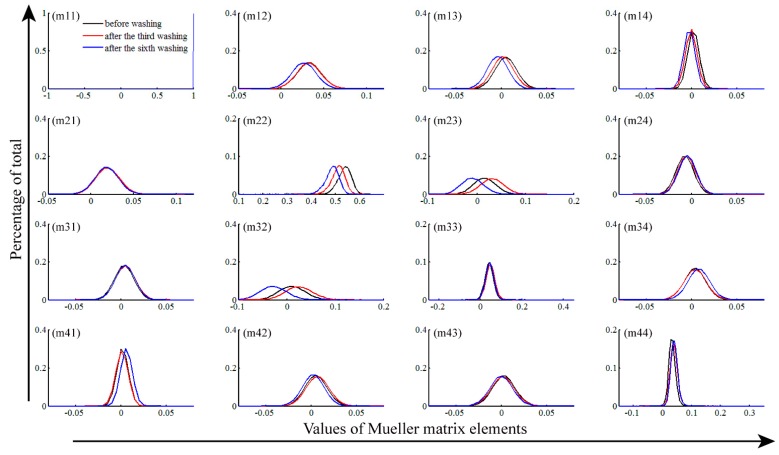
Frequency distribution histograms (FDHs) of Mueller matrix elements of silk sample washed by the fabric softener: before washing (black lines), after the third washing time (red lines) and the sixth washing time (blue lines). The areas under the FDH curves are normalized to 1, and the horizontal axis is divided into 400 parts.

**Figure 4:**
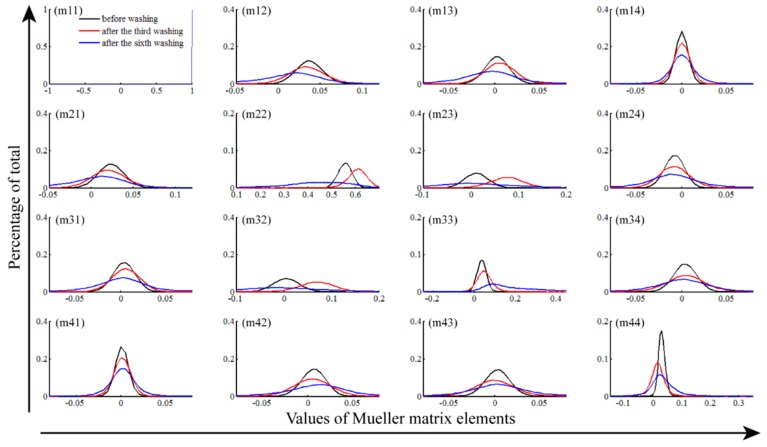
Frequency distribution histograms (FDHs) of Mueller matrix elements of silk sample washed by the color stain net: before washing (black lines), after the third wash (red lines) and the sixth wash (blue lines). The areas under the FDH curves are normalized to 1, and the horizontal axis is divided into 400 parts.

From the analysis of the 2D Mueller matrix images in Section 3.1, we can obtain a lot of information on how the wavelength-scale microstructure of the silk samples varies during the washing processes. However, these features revealed directly by the 2D images are often qualitative and sometimes too detailed. In this section, we adopt the FDH technique to transform the 2D Mueller matrix images into quantitative parameters, which can characterize the dominant wavelength-scale microstructural features of silk samples. Figure 3 and Figure 4 show the FDH curves of the 2D Mueller matrix images represented in Figure 1 and Figure 2, respectively. For more information, we also calculate the central moment parameters of the FDHs of the silk samples washed by fabric softener (Table 1), laundry powder (Table 2), toilet soap (Table 3) and color stain net (Table 4). Compared to the 2D images, the FDHs and their corresponding central moments of the Mueller matrix elements reveal the main wavelength-scale structural features of the silk samples more clearly and quantitatively [26].

The FDHs of the Mueller matrices shown in Figure 3 and Figure 4 are non-diagonal, and the curves of diagonal elements m22 and m33 are different, meaning that the silk sample is anisotropic. As the washing times increase, the difference between the FDHs of the m22 and m33 changes, indicating the anisotropy degree of the silk sample varies. For a closer observation of the anisotropy variations, we then analyze the central moment parameters shown in Table 1, Table 2, Table 3 and Table 4. It can be seen that the difference of mean values (*P1*) between the m22 and m33 varies slightly for the silk samples washed by fabric softener and laundry powder (the difference variations are 0.497 to 0.443 for Table 1 and 0.505 to 0.438 for Table 2, respectively), indicating the anisotropy degree stays relatively stable. Conversely, the difference between the *P1* values of the m22 and m33 becomes more and more obvious as the washing times increase when using toilet soap and color stain net (the difference variations are 0.521 to 0.246 for Table 3 and 0.594 to 0.259 for Table 4, respectively), showing that washing by toilet soap and color stain net causes prominent reductions in the anisotropy degree of the silk fibers. Therefore, we can divide the four types of detergents into two groups: fabric softener and laundry powder denoted as group A, toilet soap and color stain net denoted as group B. 

Figure 3 and Table 1 and Table 2 also reveal that, for the silk samples washed by group A detergents, the central moment parameters of the off-center elements stay almost the same (the maximum variation is less than 0.006). It confirms that during the washing processes, the group A detergents have limited influence on the fibrous structures of the silk samples. However, Figure 4, Table 3 and Table 4 indicate that after washing by the group B detergents, the FDHs and central moment parameters of the off-diagonal Mueller matrix elements changed more prominently. For instance, the maximum change of the *P1* value for the m12 element shown in Table 4 is larger than 0.02, demonstrating that the anisotropy degree of the silk samples has been changed by the group B detergents.

It can also be seen from Figure 4 and Table 4 that, after washing by color stain net the variance of the FDHs, or the values of parameter *P2* increase significantly. For example, in Table 4 after the sixth wash the value of *P2* for the m22 element increases more than 4 times compared to the unwashed silk sample, whereas in Table 1, Table 2 and Table 3 the values of *P2* keep relatively constant. It means the color stain net may seriously damage the well-ordered fibrous structures, distributing the fiber orientation over a wider range. We can see from Tables S1–S4 that the values of *P3* and *P4* also represent some changes, but more studies are still needed to reveal their structural meanings.

### 2.3. Mueller Matrix Parameters of Silk Sample Based on FDHs

**Figure 5:**
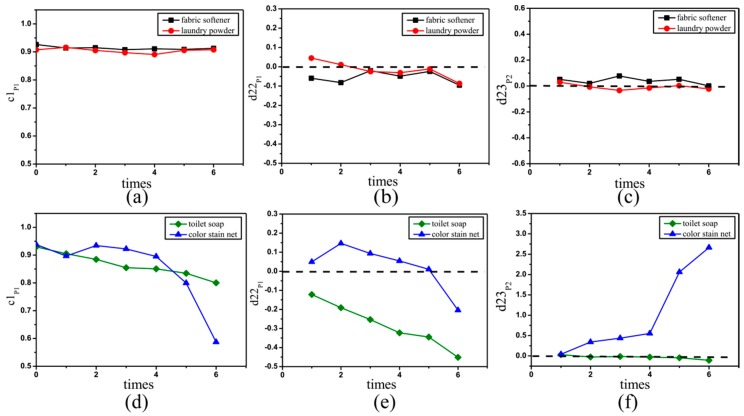
FDH parameters *c*1_p1_ (left), *d*22_p1_ (middle) and *d*23_*p*2_ (right) of silk samples washed by different detergents. (**a**–**c**) are the values of three parameters for the silk samples washed by the group A detergents: fabric softener in black square lines and laundry powder in red dot lines; (**d**–**f**) are the values of three parameters for the silk samples washed by the group B detergents: toilet soap in green diamond lines and color stain net in blue triangle lines. The horizontal axis represents the washing times.

From the figures and tables above we can see that there is abundant structural information encoded in the FDHs of Mueller matrix elements. To obtain more quantitative information on the silk from the Mueller matrix elements, we propose three new parameters based on the FDHs and their central moments. As shown in Section 2.2, the parameter *c*1_*p*1_ is an indicator for the difference between the values of diagonal m22 and m33 elements, which is positively related to the anisotropy degree of the silk sample [26]. The parameter *d*22_p1_ is an indicator for the changes of the mean value of the m22 elements, which is negatively related to the depolarization ability of the silk sample [26]. The parameter *d*23_p2_ is an indicator for the distribution width of the values of the m23 element, which is related to the degree of order of the silk sample. Figure 5 shows how the new FDH parameters *c*1_p1_, *d*22_p1_ and *d*23_p2_ vary with the washing time for the groups A (Figure 5a–c) and B (Figure 5d,e) detergents. What we can learn from Figure 5a is that, as the washing time increases, the parameter *c*1_p1_ almost remains the same around 0.9 when the silk samples are washed by the group A detergents, demonstrating that the silk samples maintain their large anisotropy degree. Moreover, Figure 5b,c show that the values of parameters *d*22_p1_ and *d*23_p2_ change mildly and fluctuate within a narrow range, indicating that the depolarization ability and degree of order of the silk samples change slightly during the washings. On the other hand, Figure 5d–f reveal that compared with the samples washed by the group A detergents, the group B detergents have more influence on the silk samples: the parameters *c*1_p1_ decreases for all samples in group B, which corresponds to reductions in the anisotropy of the silk samples. However, we also see that the other two parameters of the silk samples washed by toilet soap and color stain net have different variation features. For toilet soap, *d*22_p1_ is always negative and *d*23_p2_ decreases slightly. For color stain net, *d*22_p1_ drops from positive to negative and *d*23_p2_ increases significantly. The results shown in Figure 5 indicate that for the silk samples washed by the group B detergents, their wavelength-scale microstructures may have different characteristic variations. 

In summary we can get the following information from the analysis above: (1) The change trends of the parameters *c*1_p1_, *d*22_p1_ and *d*23_p2_ are different for the groups A and B, so they may be used to assess if a detergent affects the wavelength-scale microstructure of silk fibers; (2) In the group B, the different change trends of *d*22_p1_ and *d*23_p2_ show that the parameters may provide the details of the wavelength-scale microstructural variations of the silk samples.

### 2.4. Wavelength-Scale Microstructural Variations of Silk Samples

**Figure 6:**
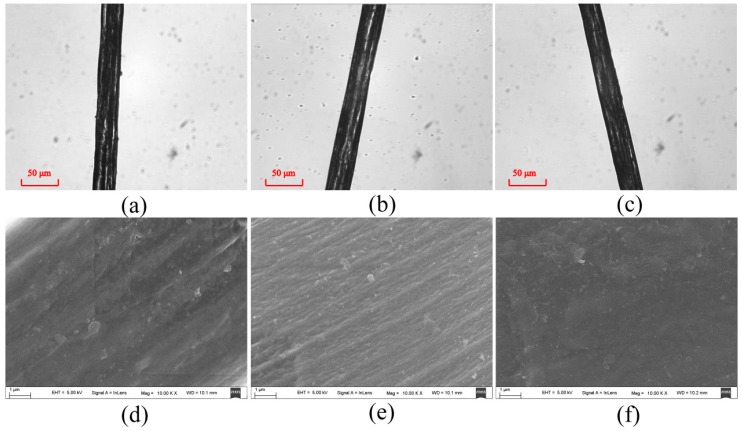
Optical microscopic images of silk samples in group A: (**a**) before washing; (**b**) after the sixth washing by fabric softener; (**c**) after the sixth washing by laundry powder. Scanning electron microscopic images (10,000×) of silk samples in group A: (**d**) before washing; (**e**) after the sixth washing by fabric softener; (**f**) after the sixth washing by laundry powder.

**Figure 7:**
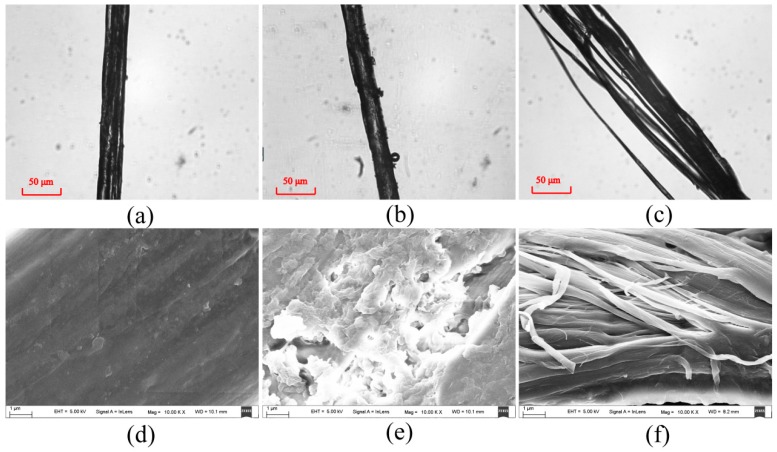
Optical microscopic images of silk samples in group B: (**a**) before washing; (**b**) after the sixth washing by toilet soap; (**c**) after the sixth washing by color stain net. Scanning electron microscopic images (10,000×) of silk samples in group B: (**d**) before washing; (**e**) after the sixth washing by toilet soap; (**f**) after the sixth washing by color stain net.

**Figure 8:**
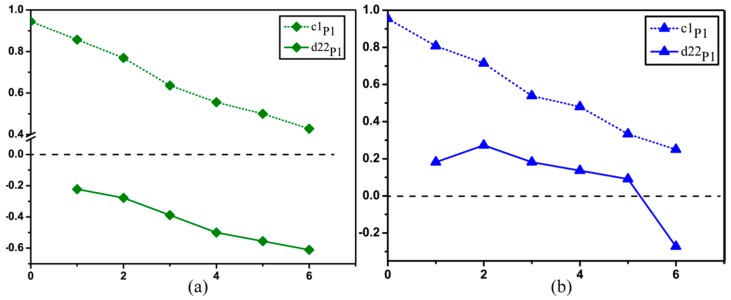
Monte Carlo simulation results of the parameters *c*1_p1_ and *d*22_p1_ using the SCSM. (**a**) The *x*-axis represents different values of sphere/cylinder ratio from (1) 10:70 to (2) 13:67; (3) 15:65; (4) 21:59; (5) 22:58; (6) 27:53 and (7) 28:52; (**b**) The *x*-axis represents different values of the diameter and standard deviation of angular distribution of the cylinders from (1) 1.5 µm 15 degree to (2) 1 µm 15 degree; (3) 0.9 µm 19 degree; (4) 0.86 µm 19 degree; (5) 0.76 µm 20 degree; (6) 0.68 µm 21 degree; and (7) 0.68 µm, 22 degree.

In order to study the relationship between the parameters used in Section 3.3 and the characteristic wavelength-scale microstructural variations of the silk samples, we observed the silk strands, which are consisted of tens of silk fibers, under an optical microscope and a scanning electron microscope. Figure 6 shows the optical and scanning electron microscopic images of the unwashed silk strand (Figure 6a,d) and the strands washed six times by group A detergents: fabric softener (Figure 6b,e) and laundry powder (Figure 6c,f). Figure 7 shows the optical and scanning electron microscopic images of silk strands before washing (Figure 7a,d) and washed six times by group B detergents: toilet soap (Figure 8b,e) and color stain net (Figure 7c,f). From Figure 6 and Figure 7, we can see that the wavelength-scale microstructures of the silk strands almost stay the same in group A, whereas they change prominently in group B. It confirms that the three new parameters based on FDHs can be used to assess the influences of the detergents on the wavelength-scale microstructures of silk samples. Moreover, in our previous discussion, we have found that for the silk samples in group B, the parameters *d*22_p1_ and *d*23_p2_ represent different change trends. This indicates that the parameters may be not only a kind of indicator to reveal whether the wavelength-scale microstructural changes occurred, but also a tool to characterize what kind of structural changes happened. It can be seen from Figure 7 that the structural changes are not the same for the silk fibers washed by toilet soap and color stain net, which demonstrates the assumption.

### 2.5. Monte Carlo Simulations

For a better confirmation of the assumption in Section 3.3, we carry out MC simulations based on the SCSM to interpret the relationships between the characteristic wavelength-scale microstructures and the Mueller matrix parameters. Since the simulated results have no obvious variance and the parameters almost do not change in group A, we focus on the analysis of the simulated parameters *c*1_p1_ and *d*22_p1_ in group B. According to the microscopic images in Figure 7 we build two models to study the contrast mechanisms of the experimental results. It can be observed from Figure 7b,e that compared to the unwashed silk fibers shown as Figure 7a,d, after six washings by toilet soap there are more bumps and burrs on the surface of the silk fibers. Therefore, we raised the sphere/cylinder ratio in the SCSM to mimic the increasing percentage of the small fragments and particles during the washing process. The parameters in the MC simulation are set as follows for the silk samples: The thickness of the medium is 0.01 cm. The diameters of the cylindrical and spherical scatterers are 1.5 µm and 0.2 µm to mimic the single silk fibers and the roughness on the silk surfaces or particles embedded among the fibers [25,27]. The refractive indices of the scatterers and interstitial medium are 1.56 and 1, respectively [25]. The cylindrical scatterers are distributed along the *x*-axis, and the standard deviation of their angular distribution is 15 degree. 

The toilet soap is alkaline, and has dehydration condensation reaction with −COOH group of amino acid. Silk is made of fibroin and sericin which are composed of amino acids. Therefore, the reactions between the silk fibers and toilet soap solution generate peptide bonds to make the microstructure of silk compact and may produce some small particles attached to the surface of silk. Meanwhile, these reactions could alter the surface structures to generate burrs and bulges. To simulate the changes in the wavelength-scale microstructures for the silk fibers after washing by toilet soap, we vary the sphere/cylinder ratio from 10:70 to 13:67, 15:65, 21:59, 22:58, 27:53 and 28:52 in the MC simulation. As shown in Figure 8a, the MC simulated parameters *c*1_p1_ and *d*22_p1_ both decrease as the washing times increase, and the values of *d*22_p1_ are always negative. The simulated results are consistent with the experimental observations shown in Figure 5. It is shown that the increasing surface roughness can reduce the anisotropy of the silk fibers, which can be reflected by the Mueller matrix parameters.

On the other hand, we can see from the microscopic images in Figure 7c,f that after washing by color stain net the large silk strand is divided into separated disordered silk fibers. The color stain net, whose primary ingredient is hydrogen peroxide H_2_O_2_ with strong oxidizing property, can oxidize protein to change the properties of the silk strands. The oxidize reactions dissolve the components of the silk, making the wavelength-scale microstructures of the silk fibers loose and disordered. In the MC simulations we reduce the diameters and increase the standard deviations of angular distribution of the cylinders at the same time, from 1.5 µm 15 degree to 1 µm 15 degree, 0.9 µm 19 degree, 0.86 µm 19 degree, 0.76 µm 20 degree, 0.68 µm 21 degree and 0.68 µm 22 degree. It can be observed that there is also a good agreement between the simulated result shown in Figure 8b and the experimental observations shown in Figure 5, meaning that during the washing process the silk strand breaks into finer fibers distributed in a wider orientation range. Based on the MC simulations using different wavelength-scale microstructural models, we can conclude that the contrast mechanism of the FDH parameters is the rising number of small particles and increasing surface roughness for the silk samples after washing by toilet soap, or the increasing disorder of the silk fibers with reduced diameters after washing by color stain net, which can be distinguished by the parameters *d*22_p1_ and *d*23_p2_ shown in Figure 5. The discovery in this study provides a potential method for characterizing the wavelength-scale microstructure of silk based materials.

### 2.6. Results of X-ray Diffraction and Discussions

The experimental and Monte Carlo simulated results shown above indicate that the Mueller matrix imaging parameters are sensitive to the wavelength-scale structural changes, which may be induced by the molecular microstructural variations resulted from the detergents with different chemical components. For confirmation, we perform the X-ray diffraction (XRD) measurements on the silk samples.

**Figure 9:**
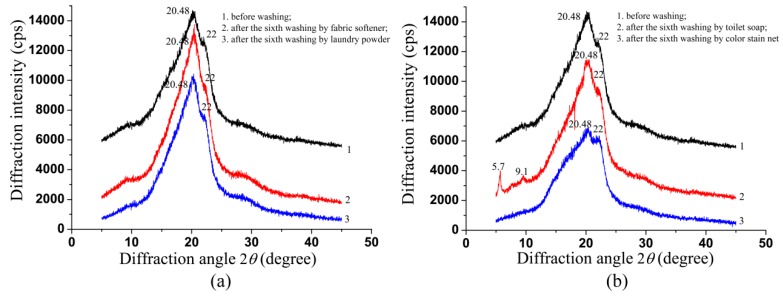
X-ray diffraction curves of silk samples. (**a**) 1. The silk before washing; 2. The silk after the sixth washing by fabric softener; 3. The silk after the sixth washing by laundry powder; (**b**) 1. The silk before washing; 2. The silk after the sixth washing by toilet soap; 3. The silk after the sixth washing by color stain net.

As shown in Figure 9a, there are no obvious differences in the diffraction peaks for the silk samples washed by group A detergents, indicating that their macromolecular microstructures almost do not change. On the other hand, Figure 9b reveals that after washings by group B detergents, the macromolecular microstructural features of the silk samples vary: (1) For the XRD curve 2, the diffraction peak near 9.1 degree is enhanced compared to the XRD curve 1. It can also be observed that there is a new diffraction peak near 5.7 degree, indicating a new conformation of the silk protein may be produced after washing by toilet soap [28]; (2) For the XRD curve 3, the diffraction peaks of 20.48 degree and 9.1 degree are obviously weakened compared to the XRD curve 1, meaning that the crystallinity of the silk protein may decrease after washing by color stain net [29]. In summary, the XRD measurements show that the group A detergents have very little influence on the macromolecular microstructures of the silk samples, while the group B detergents may alter the conformation and crystallinity of the silk protein, leading to increasing disorder and surface roughness of the silk fibers, which can be reflected by the Mueller matrix imaging parameters shown as Figure 5.

## 3. Materials and Methods

### 3.1. Experimental Setup and Silk Samples

**Figure 10:**
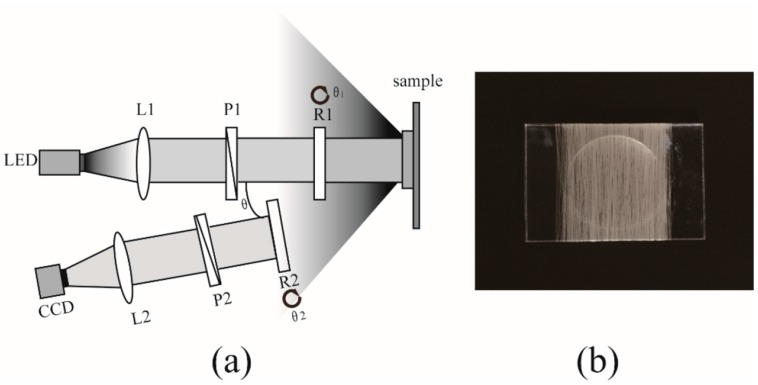
(**a**) Schematic of experimental setup for the backscattering Mueller matrix measurement. P1, P2: polarizer; R1, R2: quarter-wave plate; L1, L2: lens. The oblique incident angle θ is about 15 degree to avoid the surface reflection from the sample. The diameter of the illumination area is about 1.8 cm; (**b**) Silk sample used in this study.

We adopt a typical experimental setup based on the dual rotating retarder configuration for backscattering Mueller matrix measurements [30,31]. As shown in Figure 10a, the polarization states of the incident light from the LED (633 nm, 3 W) are controlled by a linear polarizer (P1, extinction ratio > 1000:1, Daheng Optic, Beijing, China) and quarter-wave plate (R1, Daheng Optic). Then the collimated light of different polarization states illuminates the sample, and the backscattered photons pass through another quarter-wave plate (R2, Daheng Optic) and linear polarizer (P2, extinction ratio > 1000:1, Daheng Optic). Finally the photons are collected by a CCD camera (QImaging 32-0122A, 12 bit, Surrey, BC, Canada). There is an oblique angle (θ = 15°) between the illuminating and detection arms to avoid the surface reflection from the sample.

During the measurements, the polarizers (P1, P2) are fixed in the horizontal direction, and the two retarders (R1, R2) rotate with a fixed rate θ_1_ = 5θ_2_. The Fourier series intensities are given by Equation (1).

(1) $$I=\alpha_{0}+\overset{12}{\underset{n=1}{\sum }}\left(\alpha_{n}\text{cos}\;2{n\theta }_{1}+\beta_{n}\text{sin}\;2{n\theta }_{1}\right)$$

In this paper, after 30 rotations of both retarders, the Mueller matrix elements can be calculated by using the Fourier coefficients α_*n*_ and β_*n*_ shown as Equation (1) [31]. Before applying to the samples, we calibrate the experimental setup by measuring the Mueller matrices of standard samples such as air. The maximum errors for the absolute values of all the Mueller matrix elements are smaller than 0.01 after calibration.

In order to analyze the influence of different detergents on the wavelength-scale microstructures of silk based materials, we prepared a simpler silk sample by wrapping the silk fibers (provided by Guangxi Institute of Supervision and Testing on Product Quality, Nanning, Guangxi, China) around a glass slide as shown in Figure 10b. Then the well ordered silk fibers are washed six times using four common detergents: fabric softener (Comfort, Q/YQXA306, Unilever, Hefei, China), laundry powder (Diaopai, WL-AGB/T13171.2-2009, Nice Group China, Lishui, China), toilet soap (Safeguard, Q/GZBJ8II, Procter & Gamble, Tianjin, China) and color stain net (Bluemoon, Q/LYLZG20II, Bluemoon China, Guangzhou, China). The silk fibers around the glass slide are soaked and washed in the detergent solution for 30 min. Then they are completely dried out. During the washing processes other factors are kept the same, such as the water temperature, soaking time, drying time and so on. There are no evident contractions in the silk sample. After each washing, we take the 2D backscattering Mueller matrix images of the silk samples and examine the relationship between the Mueller matrix parameters and the wavelength-scale microstructural variations of washed silk fibers.

### 3.2. Frequency Distribution Histograms (FDHs) and Quantitative Parameters

As a comprehensive description of polarization properties, Mueller matrix contains abundant structural information of samples. However, the relationships between the features of certain wavelength-scale microstructures and the Mueller matrix elements are not always clear, increasing the difficulties in applying Mueller matrix polarimetry to practical applications. To deal with this problem, one may transform the Mueller matrix elements into quantitative parameters with clearer physical meanings [32]. In this work, we use the frequency distribution histograms (FDHs) to characterize the wavelength-scale microstructural changes of silk samples [26]. Previous studies have shown that the 2D images of Mueller matrix elements of homogeneous samples can be transformed into a group of orientation insensitive parameters using FDH and its central moments, which can characterize the dominant wavelength-scale microstructural features of the samples [26]. By analyzing the peak positions, widths and shapes of the FDHs of the Mueller matrix elements, we may learn abundant information of the silk samples, such as the degree of order, depolarization power, orientation direction of the anisotropic silk fibers and so on.

In our previous studies, four central moment parameters have been adopted for the statistical analysis: mean (*P1*), variance (*P2*), skewness (*P3*) and kurtosis (*P4*) defined as Equation (2). More detailed information about the central moment parameters can be obtained according to [26].

(2) $$\begin{array}{l} \mu =P1=E\left(X\right)\\ \sigma^{2}=P2=Var\left(X\right)\\ skewness=P3=\frac{E{\left(X-\mu \right)}^{3}}{\sigma^{3}}\\ kurtosis=P4=\frac{E{\left(X-\mu \right)}^{4}}{\sigma^{4}} \end{array}$$

Suppose we have a random variable *X*, whose central moments are defined as Equation (2): mean (µ), variance (σ^2^), skewness and kurtosis. *E* and *Var* are the notations for calculating the mean (*P1* or µ) and variance (*P2* or σ^2^) values of the random variable *X*, respectively. Then, using µ and σ^2^ the skewness (*P3*) and kurtosis (*P4*) can be calculated [26]. Considering that for the backscattering Mueller matrix imaging, the central elements m22, m33, m23 and m32 always represent more prominent changes than other elements [20], we propose a new set of parameters based on their mean (*P1*) and variance (*P2*) values of the FDHs to characterize the wavelength-scale microstructure variations of the silk samples.

(3) $$\begin{array}{l} {c1}_{P1}=\frac{\left|{m22}_{P1}-{m33}_{P1}\right|}{{m22}_{P1}}\\ {d22}_{P1}=\frac{{{m22}_{i}}_{P1}-{{m22}_{0}}_{P1}}{{{m22}_{0}}_{P1}}\\ {d23}_{P2}=\frac{{{m23}_{i}}_{P2}-{{m23}_{0}}_{P2}}{{{m23}_{0}}_{P2}} \end{array}$$

As shown in Equation (3), we define the parameter *c*1_p1_ to characterize the difference between the mean values (*P1*) of the FDHs of diagonal m22 and m33. Meanwhile, the parameters *d*22_p1_ and *d*23_p2_ indicate the mean (*P1*) and variance (*P2*) differences between the unwashed and washed silk samples of the m22 and m23 elements. In Equation (3), *m*22_ip1_, *m*23_ip2_ are the mean and variance values of the FDH curves of the silk sample after the *i*th washing, while *m*22_0p1_ and *m*23_0p2_ are the corresponding values of unwashed sample. Here we choose the diagonal m22 element and off-diagonal m23 element for calculations. It should be noted that other elements can also provide similar information of the silk samples.

### 3.3. Monte Carlo (MC) Simulation

For better explanations of the relationships between the Mueller matrix parameters and the wavelength-scale microstructural changes of the silk samples, we carry out MC simulations based on the SCSM to study the behavior of polarized photons as they propagate in the silk samples [27]. There are two key components in the SCSM: spherical scatterers and infinitely long cylindrical scatterers, representing different wavelength-scale microscopic structures of the silk samples. The silk fibers are approximated as cylindrical scatterers immersed in interstitial medium, meanwhile, the small particles attached to the surface of the silk fibers or embedded in the interstitial medium can be represented by spherical scatterers with different sizes and scattering coefficients. In this paper, we set the MC simulation parameters according to the silk samples, which will be introduced in the following sections.

## 4. Conclusions

In this paper, we take the backscattering 2D Mueller matrix images of silk fibers washed by different detergents: fabric softener, laundry powder, toilet soap and color stain net. The analysis of the 2D images and frequency distribution histograms (FDHs) of the Mueller matrix elements reveals abundant qualitative structural information of the silk fibers, such as their anisotropy degree, depolarization ability and orientation direction. Moreover, we propose a group of new parameters based on the central moments of FDHs to obtain the wavelength-scale microstructural variation features of the silk samples during the washing processes quantitatively. The experimental results demonstrate that the Mueller matrix parameters have the potential to be used as indicators to decide if a detergent can change the structure of the silk fibers. Also, the different change trends show that the parameters may provide details on the wavelength-scale microstructural variations of the silk fibers. For deeper understanding of the relationship between the Mueller matrix parameters and the microstructural variations of silk, we also performed X-ray diffraction and scanning electron microscope measurements, then carried out Monte Carlo simulations based on the sphere-cylinder scattering model. The good agreement between experimental and simulated results confirms that the Mueller matrix polarimetry and FDH based parameters can be used as tools to monitor the wavelength-scale microstructural variations of silk. The information is valuable not only for the quantitative characterization of structural changes of silk fibers, but also for non-destructive and real-time detection of wavelength-scale microstructures of silk based materials.

## Figures and Tables

**Table 1:** Central moments *P1* and *P2* of the Mueller matrix elements for silk sample washed by fabric softener.

Detergent/Parameter	m12	m22	m23	m32	m33
F/P1_0	0.030	0.536	0.013	0.008	0.039
F/P1_1	0.029	0.504	0.036	0.030	0.043
F/P1_2	0.028	0.492	−0.003	−0.005	0.042
F/P1_3	0.029	0.526	0.009	0.002	0.048
F/P1_4	0.030	0.510	0.029	0.023	0.045
F/P1_5	0.029	0.524	0.015	0.009	0.048
F/P1_6	0.024	0.485	−0.012	−0.030	0.042
F/P2_0	0.011	0.022	0.018	0.022	0.016
F/P2_1	0.012	0.021	0.019	0.023	0.016
F/P2_2	0.011	0.022	0.019	0.022	0.016
F/P2_3	0.012	0.023	0.020	0.023	0.017
F/P2_4	0.011	0.022	0.019	0.023	0.017
F/P2_5	0.012	0.022	0.019	0.024	0.017
F/P2_6	0.011	0.022	0.018	0.022	0.016

**Table 2:** Central moments *P1* and *P2* of the Mueller matrix elements for silk sample washed by laundry powder.

Detergent/Parameter	m12	m22	m23	m32	m33
L/P1_0	0.032	0.528	0.075	0.071	0.049
L/P1_1	0.029	0.552	0.019	0.010	0.046
L/P1_2	0.027	0.534	0.037	0.036	0.051
L/P1_3	0.030	0.515	0.044	0.038	0.053
L/P1_4	0.032	0.512	0.056	0.050	0.056
L/P1_5	0.034	0.522	0.019	0.013	0.049
L/P1_6	0.032	0.482	0.004	−0.002	0.044
L/P2_0	0.012	0.019	0.023	0.027	0.018
L/P2_1	0.012	0.018	0.023	0.027	0.018
L/P2_2	0.012	0.018	0.022	0.026	0.018
L/P2_3	0.012	0.018	0.022	0.026	0.019
L/P2_4	0.012	0.019	0.022	0.026	0.019
L/P2_5	0.013	0.019	0.023	0.027	0.019
L/P2_6	0.012	0.019	0.022	0.026	0.018

**Table 3:** Central moments *P1* and *P2* of the Mueller matrix elements for silk sample washed by toilet soap.

Detergent/Parameter	m12	m22	m23	m32	m33
T/P1_0	0.033	0.560	0.001	−0.006	0.039
T/P1_1	0.039	0.492	0.006	−0.001	0.047
T/P1_2	0.039	0.453	−0.016	−0.023	0.052
T/P1_3	0.037	0.418	−0.004	−0.006	0.061
T/P1_4	0.033	0.379	0.003	−0.002	0.057
T/P1_5	0.031	0.367	0.004	−0.003	0.061
T/P1_6	0.026	0.307	−0.016	−0.021	0.061
T/P2_0	0.013	0.024	0.020	0.023	0.019
T/P2_1	0.013	0.025	0.021	0.023	0.019
T/P2_2	0.013	0.029	0.019	0.021	0.019
T/P2_3	0.013	0.028	0.020	0.021	0.019
T/P2_4	0.012	0.031	0.019	0.020	0.019
T/P2_5	0.012	0.027	0.019	0.020	0.019
T/P2_6	0.011	0.027	0.018	0.018	0.018

**Table 4:** Central moments *P1* and *P2* of the Mueller matrix elements for silk sample washed by color stain net.

Detergent/Parameter	m12	m22	m23	m32	m33
C/P1_0	0.034	0.555	0.009	0.002	0.034
C/P1_1	0.036	0.582	0.134	0.131	0.060
C/P1_2	0.039	0.636	0.073	0.067	0.042
C/P1_3	0.032	0.607	0.074	0.067	0.047
C/P1_4	0.030	0.585	0.017	0.006	0.061
C/P1_5	0.025	0.560	−0.010	−0.019	0.113
C/P1_6	0.010	0.441	0.003	−0.009	0.182
C/P2_0	0.013	0.024	0.020	0.022	0.018
C/P2_1	0.014	0.024	0.021	0.025	0.022
C/P2_2	0.017	0.029	0.027	0.029	0.027
C/P2_3	0.018	0.032	0.029	0.030	0.030
C/P2_4	0.019	0.040	0.031	0.034	0.036
C/P2_5	0.029	0.073	0.061	0.066	0.079
C/P2_6	0.032	0.107	0.073	0.078	0.119

F, L, T and C represent fabric softener, laundry powder, toilet soap and color stain net respectively. F/P1_0 and F/P2_0 represent the parameters *P1* and *P2* of silk sample before washing under the condition of fabric softener. The full Tables of *P1*–*P4* values of all the Mueller matrix elements are provided as supporting material.

## Supplementary Materials

Supplementary materials can be found at www.mdpi.com/1422-0067/17/8/1301/s1.
