# Supplementary material for: Characterizing the Effects of Washing by Different Detergents on the Wavelength-Scale Microstructures of Silk Samples Using Mueller Matrix Polarimetry
Source: Int J Mol Sci. 2016 Aug 10;17(8):1301. doi: 10.3390/ijms17081301 (PMC5000698; doi:10.3390/ijms17081301)
Supplement: Supplementary file 1 [file ijms-17-01301-s001.pdf]

Supplementary Materials: Characterizing the Effects of Washing by Different Detergents on the Wavelength−Scale Microstructures of Silk Samples Using Mueller Matrix Polarimetry

Yang Dong, Honghui He, Chao He, Jialing Zhou, Nan Zeng and Hui Ma

Table S1. Central moments of the Mueller matrix elements for silk sample washed by fabric softener.

Detergent/Parameter	m12	m13	m14	m21	m22	m23	m24	m31	m32	m33	m34	m41	m42	m43	m44
F/P1_0	0.030	0.002	−0.001	0.016	0.536	0.013	−0.010	0.001	0.008	0.039	0.001	−0.001	0.003	0.000	0.030
F/P1_1	0.029	0.002	−0.002	0.018	0.504	0.036	−0.011	0.002	0.030	0.043	0.000	−0.001	0.003	−0.001	0.032
F/P1_2	0.028	0.005	−0.005	0.017	0.492	−0.003	−0.008	−0.002	−0.005	0.042	−0.001	−0.000	0.005	−0.000	0.036
F/P1_3	0.029	−0.001	−0.002	0.017	0.526	0.009	−0.009	0.001	0.002	0.048	0.001	−0.001	0.003	−0.001	0.038
F/P1_4	0.030	0.001	−0.003	0.017	0.510	0.029	−0.008	0.002	0.023	0.045	0.001	−0.002	0.004	−0.001	0.037
F/P1_5	0.029	−0.002	−0.001	0.017	0.524	0.015	−0.009	0.001	0.009	0.048	0.001	−0.001	0.003	−0.001	0.038
F/P1_6	0.024	0.007	−0.005	0.016	0.485	−0.012	−0.007	0.002	−0.030	0.042	0.005	0.003	−0.001	−0.001	0.038
F/P2_0	0.011	0.009	0.005	0.011	0.022	0.018	0.008	0.009	0.022	0.016	0.009	0.010	0.010	0.010	0.009
F/P2_1	0.012	0.010	0.005	0.011	0.021	0.019	0.008	0.009	0.023	0.016	0.009	0.005	0.010	0.010	0.009
F/P2_2	0.011	0.009	0.005	0.011	0.022	0.019	0.008	0.008	0.022	0.016	0.010	0.005	0.010	0.010	0.009
F/P2_3	0.012	0.009	0.005	0.011	0.023	0.020	0.008	0.009	0.023	0.017	0.010	0.005	0.010	0.011	0.010
F/P2_4	0.011	0.009	0.005	0.011	0.022	0.019	0.008	0.009	0.023	0.017	0.010	0.005	0.010	0.010	0.010
F/P2_5	0.012	0.009	0.005	0.012	0.022	0.019	0.008	0.009	0.024	0.017	0.010	0.005	0.010	0.011	0.010
F/P2_6	0.011	0.009	0.005	0.011	0.022	0.018	0.008	0.0088	0.022	0.016	0.010	0.005	0.010	0.010	0.010
F/P3_0	0.018	0.009	0.008	0.034	−0.196	0.046	0.008	0.006	0.050	0.023	−0.020	0.001	0.028	0.009	0.152
F/P3_1	0.022	0.004	0.009	0.019	−0.090	0.021	0.002	−0.006	0.034	0.030	0.013	−0.005	0.002	−0.004	0.111
F/P3_2	0.042	0.021	−0.002	0.037	−0.251	0.010	0.000	−0.003	0.009	0.024	−0.013	0.006	−0.017	0.013	0.107
F/P3_3	0.026	0.011	0.002	0.029	−0.219	0.021	0.009	0.008	0.017	0.046	−0.015	0.014	−0.000	0.009	0.129
F/P3_4	0.036	0.004	0.007	0.039	−0.213	0.015	−0.005	0.004	0.033	0.035	0.000	−0.006	−0.001	−0.002	0.147
F/P3_5	0.033	0.004	−0.004	0.038	−0.210	0.046	−0.012	−0.001	0.035	0.038	−0.016	0.012	0.013	0.004	0.135
F/P3_6	0.021	−0.008	−0.009	0.025	−0.255	0.049	−0.007	−0.002	0.058	0.034	−0.007	0.013	−0.010	−0.000	0.138
F/P4_0	2.224	2.205	2.198	2.210	2.227	2.187	2.210	2.211	2.179	2.226	2.256	2.206	2.208	2.239	2.315
F/P4_1	2.223	2.205	2.209	2.213	2.209	2.185	2.204	2.195	2.191	2.204	2.222	2.206	2.193	2.220	2.298
F/P4_2	2.226	2.199	2.216	2.229	2.301	2.185	2.200	2.193	2.179	2.208	2.240	2.202	2.203	2.230	2.286
F/P4_3	2.211	2.195	2.200	2.218	2.249	2.199	2.223	2.201	2.187	2.210	2.244	2.215	2.211	2.237	2.312
F/P4_4	2.224	2.206	2.206	2.225	2.300	2.195	2.200	2.189	2.193	2.218	2.231	2.206	2.212	2.238	2.294
F/P4_5	2.232	2.202	2.209	2.245	2.284	2.199	2.203	2.190	2.181	2.217	2.240	2.209	2.207	2.246	2.293
F/P4_6	2.219	2.194	2.201	2.219	2.323	2.195	2.201	2.206	2.192	2.216	2.230	2.203	2.210	2.235	2.304

Table S2. Central moments of the Mueller matrix elements for silk sample washed by laundry powder.

Detergent/Parameter	m12	m13	m14	m21	m22	m23	m24	m31	m32	m33	m34	m41	m42	m43	m44
L/P1_0	0.032	0.004	−0.003	0.020	0.528	0.075	−0.011	0.003	0.071	0.049	−0.001	−0.001	0.003	−0.002	0.027
L/P1_1	0.029	0.003	0.004	0.019	0.552	0.019	−0.013	0.001	0.010	0.046	0.000	0.000	0.001	−0.004	0.032
L/P1_2	0.027	0.005	0.001	0.017	0.534	0.037	−0.008	−0.000	0.036	0.051	−0.002	0.002	−0.001	−0.011	0.036
L/P1_3	0.030	0.004	−0.002	0.020	0.515	0.044	−0.000	0.003	0.038	0.053	0.000	0.000	0.001	−0.001	0.036
L/P1_4	0.032	0.004	−0.002	0.021	0.512	0.056	−0.008	0.003	0.050	0.056	−0.001	−0.001	0.003	0.000	0.036
L/P1_5	0.034	0.002	−0.001	0.022	0.522	0.019	−0.009	0.002	0.013	0.049	0.001	−0.001	0.003	−0.001	0.034
L/P1_6	0.032	0.001	−0.006	0.021	0.482	0.004	−0.007	0.001	−0.002	0.044	0.001	−0.002	0.006	0.001	0.033
L/P2_0	0.012	0.010	0.005	0.011	0.019	0.023	0.008	0.009	0.027	0.018	0.010	0.005	0.010	0.010	0.009
L/P2_1	0.012	0.010	0.005	0.012	0.018	0.023	0.008	0.009	0.027	0.018	0.011	0.006	0.010	0.011	0.010
L/P2_2	0.012	0.009	0.005	0.012	0.018	0.022	0.008	0.009	0.026	0.018	0.010	0.005	0.010	0.011	0.011
L/P2_3	0.012	0.010	0.005	0.012	0.018	0.022	0.008	0.009	0.026	0.019	0.011	0.005	0.010	0.011	0.011
L/P2_4	0.012	0.010	0.005	0.012	0.019	0.022	0.008	0.009	0.026	0.019	0.011	0.005	0.010	0.011	0.010
L/P2_5	0.013	0.010	0.005	0.012	0.019	0.023	0.008	0.009	0.027	0.019	0.011	0.006	0.011	0.012	0.011
L/P2_6	0.012	0.010	0.005	0.012	0.019	0.022	0.008	0.009	0.026	0.018	0.011	0.005	0.010	0.011	0.011
L/P3_0	0.008	−0.007	0.006	0.013	−0.025	−0.016	−0.005	0.002	−0.044	0.040	0.005	−0.003	−0.023	−0.001	0.145
L/P3_1	0.015	0.009	0.003	0.015	−0.017	0.020	−0.012	0.013	−0.008	0.023	0.008	0.008	0.015	−0.013	0.115
L/P3_2	0.025	0.002	0.007	0.024	−0.061	0.010	−0.002	−0.003	−0.009	0.045	−0.008	0.001	−0.014	0.001	0.126
L/P3_3	0.013	−0.004	0.014	0.019	−0.044	−0.032	−0.010	0.003	−0.012	0.067	0.003	0.008	−0.001	−0.013	0.152
L/P3_4	0.018	−0.003	−0.006	0.022	−0.033	−0.036	−0.008	0.005	−0.031	0.062	−0.017	0.016	0.013	0.009	0.179
L/P3_5	0.008	0.001	0.003	0.026	−0.003	−0.041	0.007	0.004	−0.013	0.038	0.010	0.014	0.020	−0.000	0.123
L/P3_6	0.020	−0.012	−0.002	0.017	0.011	−0.038	−0.006	0.007	−0.050	0.041	−0.017	0.007	0.018	0.007	0.150
L/P4_0	2.206	2.203	2.203	2.212	2.195	2.161	2.204	2.196	2.194	2.212	2.236	2.196	2.197	2.217	2.310
L/P4_1	2.217	2.210	2.216	2.227	2.206	2.163	2.210	2.194	2.196	2.226	2.248	2.209	2.208	2.260	2.324
L/P4_2	2.222	2.206	2.209	2.220	2.208	2.212	2.201	2.200	2.246	2.219	2.245	2.207	2.205	2.249	2.307
L/P4_3	2.231	2.202	2.202	2.226	2.211	2.220	2.201	2.198	2.233	2.241	2.252	2.204	2.208	2.256	2.335
L/P4_4	2.222	2.208	2.201	2.217	2.199	2.169	2.21	2.195	2.196	2.223	2.263	2.210	2.202	2.241	2.348
L/P4_5	2.215	2.222	2.208	2.222	2.220	2.184	2.203	2.198	2.205	2.226	2.271	2.212	2.222	2.260	2.325
L/P4_6	2.225	2.212	2.217	2.235	2.207	2.174	2.214	2.212	2.204	2.224	2.268	2.212	2.209	2.280	2.334

Table S3. Central moments of the Mueller matrix elements for silk sample washed by toilet soap.

Detergent/Parameter	m12	m13	m14	m21	m22	m23	m24	m31	m32	m33	m34	m41	m42	m43	m44
T/P1_0	0.033	0.001	−0.001	0.018	0.560	0.001	−0.011	0.000	−0.006	0.039	0.001	−0.001	0.003	0.000	0.030
T/P1_1	0.039	0.002	−0.001	0.031	0.492	0.006	−0.011	0.001	−0.001	0.047	0.001	−0.001	0.003	−0.001	0.036
T/P1_2	0.039	−0.001	−0.002	0.030	0.453	−0.016	−0.009	−0.001	−0.023	0.052	0.002	−0.001	0.004	−0.001	0.041
T/P1_3	0.037	0.001	−0.000	0.030	0.418	−0.004	−0.009	−0.001	−0.006	0.061	0.001	−0.000	0.004	−0.001	0.043
T/P1_4	0.033	0.000	0.001	0.024	0.379	0.003	−0.006	−0.001	−0.002	0.057	0.003	−0.001	0.001	−0.002	0.045
T/P1_5	0.031	0.001	−0.001	0.025	0.367	0.004	−0.006	0.001	−0.003	0.061	0.001	−0.001	0.003	−0.001	0.048
T/P1_6	0.026	−0.002	−0.001	0.021	0.307	−0.016	−0.004	−0.001	−0.021	0.061	0.001	−0.001	0.003	−0.000	0.048
T/P2_0	0.013	0.011	0.005	0.012	0.024	0.020	0.009	0.010	0.023	0.019	0.011	0.006	0.011	0.011	0.010
T/P2_1	0.013	0.011	0.006	0.013	0.025	0.021	0.009	0.010	0.023	0.019	0.011	0.006	0.010	0.011	0.010
T/P2_2	0.013	0.011	0.006	0.012	0.029	0.019	0.009	0.010	0.021	0.019	0.010	0.006	0.010	0.011	0.010
T/P2_3	0.013	0.011	0.006	0.012	0.028	0.020	0.009	0.010	0.021	0.019	0.010	0.006	0.010	0.011	0.010
T/P2_4	0.012	0.011	0.006	0.012	0.031	0.019	0.009	0.010	0.020	0.019	0.010	0.006	0.010	0.011	0.010
T/P2_5	0.012	0.011	0.006	0.012	0.027	0.019	0.009	0.010	0.020	0.019	0.010	0.006	0.010	0.011	0.010
T/P2_6	0.011	0.010	0.005	0.011	0.027	0.018	0.008	0.010	0.018	0.018	0.009	0.006	0.009	0.010	0.009
T/P3_0	0.022	0.007	−0.007	0.023	−0.100	0.003	0.016	0.004	0.048	0.010	−0.018	−0.004	0.008	0.009	0.114
T/P3_1	0.017	−0.004	−0.070	0.032	−0.043	0.027	0.021	0.001	0.088	0.000	−0.003	0.007	0.016	−0.001	0.073
T/P3_2	0.027	0.000	−0.003	0.035	−0.157	−0.004	0.005	0.001	0.002	0.016	0.002	0.003	0.021	−0.011	0.104
T/P3_3	0.035	0.005	0.002	0.042	−0.084	0.018	0.011	−0.007	0.055	0.028	−0.018	0.000	0.025	0.021	0.134
T/P3_4	0.036	−0.003	−0.007	0.039	−0.047	0.003	0.012	−0.001	0.006	0.005	0.011	0.001	0.013	−0.016	0.069
T/P3_5	0.023	0.003	−0.014	0.027	−0.017	−0.005	0.006	0.008	0.013	0.016	0.001	0.007	0.005	−0.018	0.101
T/P3_6	0.033	−0.015	−0.012	0.034	−0.009	0.007	−0.003	−0.004	0.008	0.003	0.001	−0.002	0.026	−0.011	0.105
T/P4_0	2.205	2.197	2.201	2.206	2.231	2.202	2.192	2.203	2.209	2.213	2.224	2.203	2.184	2.250	2.280
T/P4_1	2.210	2.196	2.198	2.209	2.156	2.209	2.191	2.185	2.219	2.219	2.225	2.205	2.194	2.221	2.279
T/P4_2	2.211	2.193	2.194	2.217	2.226	2.199	2.197	2.197	2.213	2.219	2.218	2.199	2.202	2.220	2.301
T/P4_3	2.203	2.191	2.205	2.204	2.179	2.198	2.199	2.203	2.207	2.209	2.228	2.206	2.201	2.223	2.292
T/P4_4	2.213	2.208	2.207	2.203	2.116	2.203	2.202	2.193	2.217	2.206	2.211	2.200	2.203	2.223	2.255
T/P4_5	2.200	2.199	2.209	2.212	2.157	2.202	2.207	2.209	2.202	2.205	2.213	2.195	2.209	2.218	2.281
T/P4_6	2.199	2.203	2.199	2.205	2.148	2.206	2.192	2.194	2.194	2.207	2.204	2.195	2.204	2.207	2.257

Table S4. Central moments of the Mueller matrix elements for silk sample washed by color stain net.

Detergent/Parameter	m12	m13	m14	m21	m22	m23	m24	m31	m32	m33	m34	m41	m42	m43	m44
C/P1_0	0.034	0.000	−0.003	0.021	0.555	0.009	−0.011	0.001	0.002	0.034	0.001	−0.001	0.004	0.000	0.028
C/P1_1	0.036	0.008	−0.002	0.023	0.582	0.134	−0.015	0.006	0.131	0.060	0.000	−0.001	0.004	−0.003	0.019
C/P1_2	0.039	0.005	−0.003	0.025	0.636	0.073	−0.012	0.004	0.067	0.042	0.002	−0.001	0.002	−0.003	0.016
C/P1_3	0.032	0.003	−0.002	0.018	0.607	0.074	−0.011	0.003	0.067	0.047	0.002	−0.001	0.003	−0.003	0.013
C/P1_4	0.030	0.001	−0.002	0.017	0.585	0.017	−0.014	0.002	0.006	0.061	0.001	−0.001	0.007	−0.03	0.025
C/P1_5	0.025	−0.007	−0.002	0.013	0.560	−0.010	−0.014	−0.003	−0.019	0.113	−0.001	−0.001	0.007	0.000	0.029
C/P1_6	0.010	−0.009	−0.002	0.003	0.441	0.003	−0.012	−0.000	−0.009	0.182	−0.003	−0.000	0.008	0.003	0.031
C/P2_0	0.013	0.011	0.005	0.012	0.024	0.020	0.009	0.010	0.022	0.018	0.011	0.006	0.011	0.011	0.009
C/P2_1	0.014	0.011	0.006	0.014	0.024	0.021	0.009	0.010	0.025	0.022	0.015	0.006	0.013	0.015	0.014
C/P2_2	0.017	0.013	0.007	0.016	0.029	0.027	0.012	0.012	0.029	0.027	0.018	0.007	0.015	0.018	0.017
C/P2_3	0.018	0.014	0.007	0.017	0.032	0.029	0.014	0.013	0.030	0.030	0.019	0.008	0.017	0.020	0.020
C/P2_4	0.019	0.014	0.007	0.018	0.040	0.031	0.014	0.013	0.034	0.036	0.020	0.008	0.018	0.021	0.025
C/P2_5	0.029	0.021	0.010	0.027	0.073	0.061	0.021	0.020	0.066	0.079	0.027	0.011	0.026	0.028	0.033
C/P2_6	0.032	0.026	0.011	0.029	0.107	0.073	0.025	0.025	0.078	0.119	0.027	0.011	0.028	0.029	0.033
C/P3_0	0.013	0.002	−0.003	0.024	−0.048	0.025	−0.030	0.000	0.001	0.009	0.007	0.005	−0.056	−0.012	0.104
C/P3_1	0.139	0.041	−0.002	0.128	−0.216	−0.002	−0.037	0.016	−0.013	0.143	−0.000	0.012	0.015	−0.004	0.256
C/P3_2	0.081	0.020	−0.008	0.075	−0.028	0.031	0.008	0.013	0.030	0.094	−0.013	0.008	−0.035	−0.001	0.116
C/P3_3	0.079	0.015	−0.001	0.078	−0.145	−0.030	0.072	0.018	−0.032	0.112	−0.035	0.025	−0.010	0.030	−0.007
C/P3_4	−0.002	0.005	0.006	−0.014	−0.224	0.064	−0.052	0.012	0.081	0.288	−0.098	0.008	−0.017	0.089	0.306
C/P3_5	−0.530	−0.259	0.014	−0.433	−0.028	0.622	−0.072	−0.119	0.518	1.152	−0.194	0.107	−0.324	0.065	0.524
C/P3_6	−0.423	−0.099	0.143	−0.383	−0.305	−0.001	0.085	0.073	−0.025	0.794	−0.156	0.033	−0.182	0.149	0.345
C/P4_0	2.224	2.210	2.208	2.217	2.194	2.194	2.194	2.215	2.196	2.196	2.242	2.214	2.205	2.235	2.308
C/P4_1	2.337	2.226	2.249	2.327	2.337	2.243	2.263	2.222	2.227	2.323	2.517	2.247	2.270	2.490	2.576
C/P4_2	2.252	2.223	2.235	2.231	2.219	2.195	2.251	2.224	2.208	2.299	2.385	2.230	2.249	2.379	2.520
C/P4_3	2.279	2.220	2.234	2.281	2.319	2.252	2.279	2.222	2.245	2.350	2.472	2.246	2.267	2.465	2.585
C/P4_4	2.288	2.234	2.233	2.268	2.317	2.268	2.322	2.230	2.281	2.436	2.505	2.233	2.292	2.486	2.762
C/P4_5	3.150	2.766	2.545	3.023	2.590	3.428	2.700	2.732	3.149	3.912	2.880	2.646	2.849	2.827	2.970
C/P4_6	2.602	2.539	2.489	2.574	2.309	2.490	2.656	2.670	2.386	2.740	2.610	2.487	2.538	2.635	2.751

F, L, T and C represent fabric softener, laundry powder, toilet soap and color stain net respectively. F/P1_0 represents the parameter P1 of silk sample before washing under the condition of fabric softener, and by analogy, C/P4_6 represents the parameter P4 of silk sample after the sixth washing by color stain net.
